# Ventilator-Associated Pneumonia Caused by Multidrug-Resistant Gram-Negative Bacteria in Vietnam: Antibiotic Resistance, Treatment Outcomes, and Colistin-Associated Adverse Effects

**DOI:** 10.3390/healthcare10091765

**Published:** 2022-09-14

**Authors:** Thu Pham Minh Vo, Thien Chi Dinh, Hung Viet Phan, Thuy Thi My Cao, Phuoc Thien Duong, Thang Nguyen

**Affiliations:** 1 Department of Internal Medicine, Can Tho University of Medicine and Pharmacy, Can Tho City 900000, Vietnam; 2 Department of Pediatrics, Can Tho University of Medicine and Pharmacy, Can Tho City 900000, Vietnam; 3 Department of Respiratory, Can Tho Central General Hospital, Can Tho City 900000, Vietnam; 4 Intensive Care Unit, Can Tho Central General Hospital, Can Tho City 900000, Vietnam; 5 Department of Pharmacology and Clinical Pharmacy, Can Tho University of Medicine and Pharmacy, Can Tho City 900000, Vietnam

**Keywords:** pneumonia, ventilator-associated, drug resistance, multiple, gram-negative bacteria, colistin, Vietnam

## Abstract

Background: Ventilator-associated pneumonia (VAP) caused by multidrug-resistant (MDR) gram-negative bacteria (GNB) presents a serious clinical scenario, and there is disagreement regarding the role of colistin in treatment. This study aimed to characterize the antibiotic resistance of MDR GNB and evaluate the treatment outcomes and side effects of colistin in VAP patients caused by MDR GNB, particularly in Vietnam. Methods: A prospective cohort research was undertaken. We enrolled 136 intubated patients diagnosed with VAP according to the Centers for Disease Control and Prevention (CDC) 2019. Sixty-six individuals with an isolated gram-negative bacterium (*Acinetobacter baumannii*, *Klebsiella pneumoniae*, or *Pseudomonas aeruginosa*) satisfied the European Centre for Disease Prevention and Control (ECDC)’s criteria for multi-antibiotic resistance. Results: GNB resistance was categorized as 10.6% MDR, 63.6% XDR, and 25.8% PDR. GNB were resistant to β-lactams 80–100%, aminoglycosides 50–86.7%, fluoroquinolones 100% and colistin 2.8–20%. The 28-day mortality rate was 54.5%, and acute kidney injury occurred at 12.1%. There was no statistically significant difference in mortality rate between groups receiving regimens with or without colistin (58.3% and 73.3%, respectively; OR = 1.964; 95%CI 0.483–7.989). Neither was there a statistically significant difference in acute renal damage rate between groups receiving regimens with or without colistin (14.3% and 9.7%, respectively; OR = 1.556; 95%CI 0.34–7.121). Conclusions: GNB had a high rate of antibiotic resistance to most antibiotics. The addition of colistin to the medication did not show significant differences in renal toxicity or mortality, while colistin resistance was relatively low; larger studies need to be conducted.

## 1. Introduction

Due to the possibility of multidrug-resistant (MDR) bacterial infection, ventilator-associated pneumonia (VAP) has been identified as a severe disorder with a high fatality rate. Pneumonia is relatively common in mechanically ventilated patients—around 45% of individuals who receive mechanical ventilation for more than 5 days acquire pneumonia [1]. Patients with VAP require a more extended stay in the intensive care unit (ICU) and more intravenous antibiotics than patients without VAP due to the indications to start VAP. Treatment of VAP patients involves a cocktail of antibiotics, which increases mortality, treatment regimen toxicity, and cost burden. VAP is expected to have a mortality rate of 10 to 40% [1]. Antibiotic resistance is a global health problem and one of today’s most critical threats. Certain bacteria strains are practically immune to all antibiotics. The World Health Organization (WHO) produced a list of the top agents of antibiotic resistance in 2017, highlighting the critical need for new antibiotics, particularly for gram-negative bacteria (GNB). Due to their unique structure, GNB are more resistant to antibiotics than gram-positive bacteria, which contributes significantly to global morbidity and death [2]. GNB comprise around 65% of the agents responsible for VAP in the ICU. Patients with VAP caused by GNB suffer severe symptoms due to the bacteria’s multi-antibiotic resistance. In the Asia-Pacific area, more than half of the agents causing lower respiratory tract infections caused by GNB are extensively drug-resistant (XDR) or pan drug-resistant (PDR) [3]. Thus, effectively treating MDR infections requires eradicating a significant reservoir of infection in clinical practice. Moreover, the combination of colistin with different regimens is considered the final resort for reducing mortality in patients with VAP caused by MDR GNB, such as *A. baumannii*, *K. pneumoniae*, or *P. aeruginosa.* They have failed to respond to early treatment. Additionally, in prior studies, the outcome of VAP was better in patients who received inhaled colistin with intravenous colistin than those who received intravenous colistin alone [4,5,6]. However, worldwide studies have documented colistin resistance, and there is disagreement over the efficacy and adverse effects of the colistin combination in treating VAP [6,7,8]. Additionally, there are few studies in Vietnam on infections caused by multi-resistant GNB and the use of colistin in combination with other antibiotics. This classification restricts access to microbiological data in nosocomial infections and makes selecting a suitable antibiotic regimen for patients infected with MDR GNB in Vietnam more challenging. As a result of this state of affairs, and to gain a better understanding of the recent antibiotic-resistant characteristics of MDR GNB strains, as well as to standardize empiric antibiotic regimens, we conducted a study on patients with VAP caused by MDR GNB with the following objectives: (1) Determining antibiotic resistance characteristics of MDR GNB causing VAP; (2) Evaluating treatment outcomes, colistin side effects, and related factors in patients with VAP caused by MDR GNB.

## 2. Materials and Methods

### 2.1. Study Designs and Sampling Methods

A prospective observational study was conducted in the ICU at Can Tho Central General Hospital—a hospital in Southern Vietnam, between 3/2019 and 5/2021. Patients chosen were those aged over 18 years old, who matched the CDC 2019 criteria for VAP: (1) mechanical ventilation ≥ 48 h after intubation, (2) the appearance and/or progression of infiltrative lesions on chest X-ray, (3) the presence of at least two of the three criteria (fever ≥ 38 °C or hypothermia < 36 °C, leukocytosis ≥ 12,000/mm^3^ or leukopenia < 4000/mm^3^, and turbidity and/or an increase in the amount of endotracheal-aspirated secretions); isolated agent being one of the listed MDR GNB: (1) the number of bacteria isolated in cultures exceeded the microbiological diagnostic threshold (10^5^ CFU/mL for bronchial fluid aspirated through an endotracheal tube, 10^4^ CFU/mL for broncho-alveolar lavage, and 10^3^ CFU/mL for protected bronchial brush), (2) the organism belonged to one of the listed GNB (*A. baumannii*, or *K. pneumoniae*, or *P.s aeruginosa*), (3) being a multi-resistant organism classified by the ECDC (MDR (insensitive to ≥1 antibiotic in ≥3 antibiotic classes in the antibiogram), or XDR (insensitive to ≥1 antibiotic in all antibiotic classes, but still sensitive to ≤2 tested classes), or PDR (insensitive to all antibiotics of all classes tested)). Patients were excluded from the study if they had: (1) pneumonia within 48 h of intubation, (2) a culture of lower respiratory tract secretions that did not develop bacteria, a bacterial count that did not meet the microbiological diagnostic threshold, or agents that did not belong to one of the three agents studied (*A. baumannii*, *K. pneumoniae*, or *P. aeruginosa*), (3) pregnancy, postpartum, (4) currently receiving immunosuppressive therapy, cytotoxicity, (5) no consent of his/her relatives to participate in the study. We estimated the sample size using the proportional estimation formula: ${n=Z}_{1\;-\;\frac{\alpha }{2}}^{2}\cdot \frac{p(1\;-\;p)}{d^{2}}$, where *α* = 5% and *d* = 0.09; *p*_1_ = 0.88 represents the resistance rate of GNB to ciprofloxacin in patients with VAP, as reported in a study by Phuc L.H., Dung T.N., and Yen T.K.H. (2019) [9], so the estimated sample size *n*_1_ = 50 and *p*_2_ = 0.857 represents the clinical cure rate in patients with pneumonia caused by multi-resistant *P. aeruginosa* treated with meropenem combined with colistin, as reported in a study by Falagas et al. (2010) [10], so the estimated sample size *n*_2_ = 59. As a result, the study’s minimum estimated sample size is *n* = 59. Indeed, we gathered 66 samples.

### 2.2. Data Collection and Analysis

Samples were chosen arbitrarily. The past medical history, medical history, and paraclinical test findings were obtained from medical records, while clinical indicators were assessed directly. For objective 1, we calculated the rate of multi-resistance classification of GNB, including MDR, XDR, and PDR, using the antibiogram. Additionally, the resistance rate was assessed for each antibiotic or antibiotic subclass. For objective 2, the following contents are evaluated: the rate of regimens prescribed empirically when a patient is newly diagnosed with VAP, the rate of regimens containing carbapenem or piperacillin/tazobactam, and the consistency of the regimen when compared to the ATS/IDSA 2016 recommendations and the antibiogram results. If the patients had good clinical conditions (considered as the patient’s consciousness improved, the temperature came back to normal range, the number of leucocytes tended to be in the normal range, or the oxygenation improved), the initial regimen was continued, or a different regimen was utilized instead (referred to as the alternative regimen) based on the antibiogram if their clinical courses were unfavorable. The fraction of regimens containing colistin was determined for alternate regimens.

**Figure 1:**
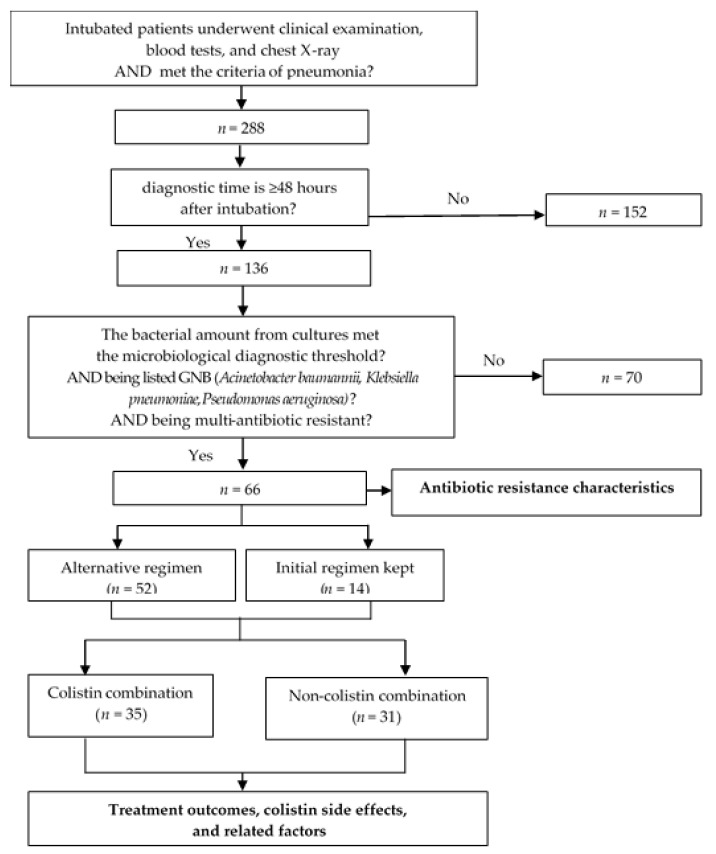
Study diagram.

The average duration of therapy and method of administration was used to illustrate the characteristics of the colistin combination. The following parameters were used to assess the effectiveness of treatment for VAP induced by multi-resistant GNB: response rate to the initial regimen, 7-day mortality rate, and 28-day mortality rate. Additionally, we examined the association between the 28-day treatment results and the initial regimen’s consistency with the change in antibiotic regimen, the colistin + carbapenem combination style, and the method of colistin administration. The incidence of acute renal injury-also known as acute kidney injury was used to assess therapeutic side effects. According to Acute Kidney Injury Network (AKIN) 2016, acute renal injury was determined when serum creatinine doubled, or eGFR decreased by more than 50% from the creatinine level before treatment in patients with normal renal function-eGFR ≥ 50 mL/min/1.73 m^2^. In patients with prior impaired renal function-eGFR < 50 mL/min/1.73 m^2^, this event was determined when serum creatinine increased >50% or eGFR decreased by more than 20% from baseline or the requirement of renal replacement therapy. The changes in serum creatinine must occur for at least 2 consecutive days and appear earliest on the second day after using antibiotics. The association between this adverse event and colistin was investigated, along with the route of colistin administration (Figure 1).

### 2.3. Statistical Analysis

Statistical Package for Social Sciences (SPSS) 18.0 by the International Business Machines (IBM) Corporation, Armonk, NY, USA, and Microsoft Excel 15.0 released in 2013 by Microsoft Corporation, One Microsoft Way, Redmond, WA, USA, were used to enter, manage and analyze data. Quantitative data with normal distribution had the mean and standard deviation calculated, whereas qualitative variables had frequency and percentage. The *t*-test was used to compare two means, and the χ^2^ test (with Fisher’s correction for tables with less than five counts) was developed to compare two proportions. Multiple logistic regression was applied to correct for possible confounding factors related to the 28-day mortality rate, whereby variables with *p* < 0.2 in the bivariate analysis were included in the model. All analyses were considered statistically significant at *p* < 0.05.

### 2.4. Ethics Approval

This study has been accepted by the Medical Ethics Committee of Can Tho University of Medicine and Pharmacy and the central hospital with decision No. 354/QD-DHYDCT on 20 February 2019.

## 3. Results

### 3.1. Study Population Characteristics

General characteristics stratified by antibiotic resistance classification of multi-resistant GNB showed that the population is comparable (Table 1 and Table 2). *A. baumannii* made up nearly 70% of GNB. Most of the patients experienced fever and increased endotracheal secretion volume. Rales were found in over 90% of the individuals. Most patients exhibited leukocytosis and low blood oxygen levels. Chest X-rays showed indistinct infiltrates around hila or multiple spots (Table 3).

### 3.2. Antibiotic Resistance Characteristics

In the study, XDR and PDR categories were the most common. *A. baumannii* was over 90% resistant to β-lactams and 75% resistant to aminoglycosides, and colistin resistance was less than 3%. More than 80% of *K. pneumoniae* were resistant to β-lactams and more than 50% to aminoglycosides. This bacterium’s antibiogram did not include colistin. *P. aeruginosa* was 100% resistant to β-lactams, 80% resistant to aminoglycosides, and 20% resistant to colistin. All three bacteria had 100% fluoroquinolone resistance (Table 4).

### 3.3. Treatment Outcomes, Colistin Side Effects, and Related Factors

Most patients received two antibiotics initially. Subgroups carbapenem and fluoroquinolone are commonly utilized in empiric regimens; 60/66 initial antibiotic regimens showed consistency with the recommendation ATS/IDSA 2016 guidelines. Compared to the antibiogram results, 62/66 (94%) antibiotic cases appeared inconsistent. Fifty-two out of these 66 (79%) patients had their regimens modified, and most of the new combinations included carbapenem; 35 of 52 (67%) of alternate regimens contained colistin. Colistin was given for a mean of 10.7 days, virtually exclusively intravenously (Table 5).

Although the initial treatment response was 65.2%, 28-day mortality was 54.5%. Acute kidney injury occurred in 12.1% of subjects (Table 6).

Underlying diseases, fever, rales, decreased oxygenation rate, and bilateral infiltrates on chest X-rays did not show differences in mortality in VAP patients. Among patients with *A. baumannii,* 28-day mortality was higher than those with other GNB but not significantly. A decrease in mortality rate was observed when the original regimens were consistent with the ATS/IDSA 2016 recommendations or the antibiogram results. However, this decrease was not statistically significant. The patients who stayed on original antibiotic regimens had a higher survival rate than those utilizing alternate regimens (*p* = 0.005). Despite no statistical significance, the carbapenem + colistin combination had a greater survival rate than other antibiotic combinations (Table 7).

**Figure 2:**
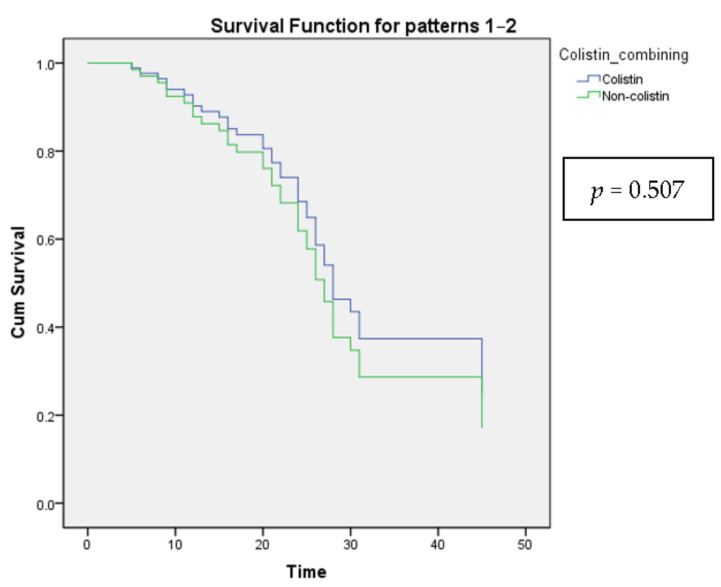
The Cox proportional-hazards model shows the survival curves for all categories of the categorical variable Colistin combining.

Leukocytosis showed a decreased 28-day mortality rate, while fever led to an increase after being tested by multiple logistic regression (Table 8). However, there was no significant difference when analyzed by Cox proportional-hazards regression (Table 9 and Figure 2).

It was not statistically significant that participants receiving colistin-containing regimens or nebulization-adjuvant-to-intravenous-colistin therapy had a more acute renal damage rate (Table 10).

## 4. Discussion

In our investigation and others, *A. baumannii* accounted for the majority of three multi-resistant GNB. In our investigation, *A. baumannii*, *K. pneumoniae*, and *P. aeruginosa* were detected at rates of 68.2%, 22.7%, and 9.1%, similar to the rates in a study by Phuc H. L. (68.2%, 18.3%, and 13.5%, respectively) [9]. Outside of Vietnam, *P. aeruginosa* was more prevalent than *K. pneumoniae*. There were 41.6% *A. baumannii*, 22.6% *K. pneumoniae*, and 35.8% *P. aeruginosa* in Bonnell’s study (χ^2^ = 24.362, *p* < 0.001) [11]; 61.5%, 7.7%, and 30.8%, respectively, in Gupta’s study (χ^2^ = 29.934, *p* < 0.001) [12] and 76%, 5.8%, and 18.2%, respectively, in Korbila’s study (χ^2^ = 36.145, *p* < 0.001) [4]. The disparities across the studies could be explained by geographical variances, differences in the medical environment, and differences in the characteristics of the patients.

Our study’s isolation rates of multi-resistant GNB were 10.6% MDR, 63.6% XDR, and 25.8% PDR. However, a 2014 report by Ninh V. D. revealed a disparity—of 22.9% MDR, 77.1% XDR, and 0% PDR [13].

*A. baumannii* was highly resistant to β-lactams: piperacillin/tazobactam 97%, aztreonam 100%, ceftazidime 97%, cefepime 97%, imipenem 97%, meropenem 97%. The resistance rate of *A. baumannii* to ceftazidime, cefepime, imipenem, and meropenem was similar in Tinh H.L.’s study [14]. In Nowak’s study, resistance to imipenem and meropenem was similar [15]. In our investigation, *A. baumannii* was resistant to amikacin (78.6%), gentamicin (86.7%), and tobramycin (84.2%). Phuc H. L. found similar resistance rates to amikacin (91%), gentamicin (80.2%), and tobramycin (74.4%) [9]. In our study, resistance rates to amikacin and tobramycin were similar to the rates in Nowak’s study (86,1% and 78,5%, respectively) [15]. *A. baumannii* in our study was 100% resistant to fluoroquinolones, which was compared to the rates in Tinh H.L.’s study (98%) [14], in Nowak’s study (98.5%) [15], and in Phuc H. L.’s study (91.8–95.3%) [9]. Our study found 2.8% resistance to colistin. This rate is similar to Phuc H.L.’s study (12%) [9]. *A. baumannii* in Tinh H.L.’s study was not resistant to colistin (*p* < 0.001) [14], while the resistance of *A. baumannii* to colistin in Nowak’s study was 47% (*p* < 0.001) [15]. The differences across studies could be explained by variations in regional characteristics, medical environments, or antibiotic usage characteristics.

The resistance rate of *K. pneumoniae* to piperacillin/tazobactam was 93.3% in our study but was significantly lower in Bandic-Pavlovic’s study (55%, *p* = 0.003) and Phuc H. L’s study (47.8%, *p* = 0.001) [9]. Most of the remaining β-lactam medicines were 100% resistant to *K. pneumoniae*, except for imipenem, which was 86.7% resistant. Meanwhile, Phuc H.L.’s research revealed that *K. pneumoniae* was resistant to ceftazidime at 78.3%, cefepime at 73.9%, and imipenem at 47.8% (*p* = 0.003) [9]. *K. pneumoniae* was 50% resistant to amikacin. This rate was slightly higher than the rate reported in Phuc H.L.’s study (22.8%) but not statistically significant [9]. Our investigation demonstrated that *K. pneumoniae* was completely resistant to ciprofloxacin and levofloxacin. Colistin resistance was not detected in our study’s antibiogram samples of *K. pneumoniae*. In Bandic-Pavlovic’s investigation, the rate of ciprofloxacin resistance was 55% [16]. In Phuc H.L.’s study, ciprofloxacin resistance was 69.6%, levofloxacin resistance was 56.5%, and colistin resistance was 30.8% [9]. An explanation for the variations in studies could be changes in antibiotic usage during each period, differences in geographical characteristics, or medical environments.

*P. aeruginosa* was resistant to all β-lactam antibiotics tested in our study (piperacillin/tazobactam, aztreonam, ceftazidime, cefepime, imipenem, and meropenem). According to Bandic-Pavlovic’s research, *P. aeruginosa* was resistant to piperacillin/tazobactam at 68%, imipenem at 74%, and meropenem at 68% [16]. Resistance to piperacillin/tazobactam was 58.8%, ceftazidime was 64.7%, cefepime was 64.7%, imipenem was 64.7%, and meropenem was 70.6%, according to Phuc H.L.’s research [9]. In our investigation, amikacin resistance occurred at an 80% rate. This rate was comparable to that observed in the Phuc H.L. study (58.8%) [9], as well as in the Pérez study (37.7%) [17]. Compared to other trials, the resistance rates of *P. aeruginosa* to the remaining antibiotics in the aminoglycoside group were similar. In our investigation, the resistance rate of *P. aeruginosa* to gentamicin was 83.3%, which was compared to the rates in Phuc H.L.’s study (64.7%) [9] and Pérez’s study (49%) [18]. Resistance to tobramycin was 80% in our study, 64.7% in Phuc H.L.’s study [9], and 41.5% in Pérez’s study [17]. In our investigation, *P. aeruginosa* was completely resistant to fluoroquinolones. According to Phuc H.L.’s research, this rate was 76.5% for ciprofloxacin and 82.4% for levofloxacin, according to Phuc H.L.’s research [9]. Pérez’s investigation revealed a reduced rate of resistance to ciprofloxacin (54.7%) and levofloxacin (58.5%) [17]. In our investigation, *P. aeruginosa* exhibited a 20% resistance rate to colistin. This percentage was comparable to the rate observed in Pérez’s study (5.7%) [17], although Phuc H.L.’s investigation revealed no colistin resistance (*p* < 0.001) [9].

When diagnosed with VAP, most patients received a combination of two or three medicines, with a combination of two antibiotics accounting for 80.3% of cases; 13.6% of patients received monotherapy. This characteristic was similar in our study to Huy B.N. (the rate of combination regimens was 94.5%, particularly the rate of two antibiotics regimens was 92.7%) [18]. Carbapenem was the most frequently used antibiotic in the early regimens, accounting for 57.6% of cases. According to a study by Quyen T.X.P. (62.3%), this rate was comparable to the rate of carbapenem used in the first treatment. Carbapenem has been critical in treating severe infections, particularly those requiring intensive care. Due to carbapenem’s broad-spectrum antibacterial characteristics, it has been recommended as an empirically initial antibiotic for VAP and other severe gram-negative infections in the ICU while waiting for microbiological results [19].

Antibiotics used in the first regimens in patients with VAP were compared to the empiric antibiotic regimens advised by ATS/IDSA 2016 (in the absence of microbiological results) and to antibiogram results to determine consistency. Although our study achieved greater than 90% compliance with the ATS/IDSA 2016 recommendations, it demonstrated inconsistency with antibiogram results, with a high rate (93.9%). This aspect was explained by our study focused on patients with VAP caused by MDR GNB, with roughly 90% of cases caused by XDR or PDR bacteria. As a result, bacteria are resistant to practically all antibiotics when tested using the antibiogram method. As a result, while conforming to the ATS/IDSA 2016 recommendations, the antibiotics currently being utilized had resistance results, resulting in contradictions with the antibiograms. The Huy B.N. study likewise produced similar results. The initial regimens were 87.3% and 14.5%, consistent with the ATS/IDSA 2016 recommendations and the antibiograms’ results in this author’s study, respectively [18]. The antibiogram discrepancy percentage was similar to that found in Quyen T.X.P.’s study (96.7%, *p* = 0.209) [20]. This similarity can be explained by research conducted in medical settings that share numerous characteristics regarding treatment and resistance to pathogenic microorganisms.

Due to the severity of the disease caused by multi-resistant bacteria, up to 78.8% of patients with VAP in our study had their treatment regimen changed due to an unsatisfactory clinical course. Carbapenem remained the most frequently utilized component in those alternate regimens, accounting for 75% of the regimens. This finding was consistent with Huy N.B.’s observation that 71% of alternate regimens comprised carbapenems [18]. Additionally, we discovered that, in various regimens with or without carbapenem, colistin remained the most often used for combination (with 61.5% of carbapenem-containing regimens and 84.6% of non-carbapenem-containing regimens). The choice of colistin over aminoglycosides or other antibiotics may be connected to the resistance features of the microorganisms in our investigation. Indeed, the antibiogram of multi-resistant GNB revealed that *A. baumannii* was resistant to colistin at a rate of 2.8%, and *P. aeruginosa* was resistant at a rate of 20%, while resistance to fluoroquinolones (100%) and resistance to amikacin (50–80%) was extremely high.

The effectiveness of treatment was determined by the rate of response to the initial regimens, the rate of mortality, and the rate of acute renal damage during treatment.

In our study, 65.2% of individuals responded to the first regimens. This percentage was comparable to Cisneros’s (75.8%) [21], Oanh T.N.P.’s (57.7%) [22], and Korbila’s (72.7%) [4]. In Karakuzu’s trial, the percentage of participants who reacted well to initial regimens was much lower (49.1%, χ^2^ = 6.804, *p* = 0.009) [23]. This could be explained by the studies using different response definitions to empirically initial antibiotic therapy.

The 28-day mortality rate in our study was 54.5%. This rate was comparable to the 52.9% of Oanh T.N.P.’s study [22]. Meanwhile, some studies found a lower death rate, such as Cisneros’s (30.6%, χ^2^ = 17.82, *p* < 0.001) [21] and Karakuzu’s (25.1%, χ^2^ = 30.439, *p* < 0.001) [23]. This disparity may be explained by the differences in patient characteristics related to the underlying diseases, risk factors, and prognosis of VAP and the variation in scientific-technical progress, antibiotic control, and MDR microorganisms.

Acute renal damage occurred in 12.1% of patients in our study. This is much less than the rate observed in Huy H.N’s study (24.5%, χ^2^ = 5.467, *p* = 0.019) [18]. This discrepancy could be explained by differences in patient characteristics and intervention procedures because, in addition to the use of toxic drugs, other factors, such as dehydration, infections, particularly shock, multi-organ failure, etc., could also be the primary cause or contribute to the acute kidney injury [24].

The presence of leukocytosis was associated with lower mortality, which may be in contrast to data in published works. The small sample size may explain this, and studies with a larger sample size should be conducted to investigate this point.

Patients who remained on the original regimen had a mortality rate of 21.4%, three times lower than those who had the regimen changed. Patients in good clinical conditions should stay on current antibiotic regimens. In contrast, patients who required regimen changes had severe illness features, which predicted greater treatment challenges than patients who made satisfactory clinical progress. Due to the favorable clinical courses, this group of patients who stayed on current antibiotic regimens had a greater survival rate than those utilizing alternate regimens. Alternative regimens were developed to assist if the initial regimens were ineffective, and the patient encountered numerous challenges throughout therapy. Numerous alternate regimens have been shown to improve treatment outcomes to varying degrees. In our investigation, carbapenem was the active substrate frequently used in alternate regimens. Additionally, colistin is commonly used with other agents in alternate regimens. As a result, we evaluated the benefit of the carbapenem-colistin combination. Although the results were not statistically significant, they suggested that carbapenem + colistin improved treatment outcomes by lowering mortality compared to other combinations. When analyzing the efficacy of colistin combination therapy, it was also necessary to evaluate the route of colistin administration. Although nebulization-adjuvant-intravenous colistin therapy resulted in a reduction in mortality, this reduction was not statistically significant. According to a meta-analysis of colistin’s involvement in treating VAP caused by MDR GNB, nebulization-adjuvant-intravenous colistin therapy improved clinical recovery. Still, it did not affect mortality compared to solely intravenous colistin therapy [8].

The combination of colistin in alternative regimens could be beneficial in treatment. However, adverse events on kidneys were possible with antibiotics, including colistin. In our study, the rate of acute kidney injury appeared more in patients using colistin components than in regimens without colistin. However, it was impossible to conclude that colistin increased the incidence of acute kidney injury because the difference was not statistically significant. Florescu’s study also showed similarities to this rate [7]. We also investigated whether colistin nebulization adjuvant intravenous therapy was associated with an increased incidence of acute kidney injury than intravenous colistin alone. A meta-analysis and our study concluded no difference in the incidence of acute kidney injury in the two mentioned therapy groups [8].

## 5. Strengths and Limitations

One of the study’s strengths is investigating the resistance rate to multi-resistant GNB in Vietnam, with frightening results. Additionally, the study discovered that fluoroquinolone was one of the two most often utilized antibiotic subgroups in the initial regimens, even though all three GNB were resistant to this antibiotic 100% of the time. GNB exhibited a prevalence of resistance to colistin. However, the combination of colistin in alternate regimens was suggested due to the study’s positive clinical outcomes, with no significant difference in the incidence of renal adverse events. After all, the innovation point in the study is that it has been representative of Vietnam, especially the South, where studies of GNB infections were still limited. The study has provided updated data on antibiotic resistance characteristics of this bacteria group and takes part in restarting studies that focus on the role of colistin in treatment, including the effectiveness and toxicity of various application modes in the future.

This study may have several limitations. To begin, our investigation was conducted in a single center. Perhaps it is important to duplicate the research model in additional health facilities to ensure it is typical of Vietnam’s South. Second, there was no colistin tested in *K. pneumoniae*’s antibiograms. This could influence the general resistance rate, the guide for choosing antibiotics for alternative regimens. Third, no control group was established at the start of the trial, and we kept track of whether or not the group received colistin throughout our trial. Fourth, this study’s assessment of colistin’s adverse effects focused exclusively on a renal event. The investigation is not yet complete, particularly regarding renal adverse effects.

## 6. Conclusions

The majority of cases were classified as MDR or PDR. GNB had a high resistance rate to most antibiotics. There were no statistically significant changes in mortality or the rate of acute renal injury between alternate regimens with or without colistin or between colistin administration routes, while colistin resistance was relatively low; more studies need to be conducted further.

## Figures and Tables

**Table 1:** Antibiotic resistance classification of multi-resistant GNB (*n* = 66).

Resistance Classification, *n* (%)	
MDR	7 (10.6)
XDR	42 (63.6)
PDR	17 (25.8)

**Table 2:** General characteristics of the patients in the study stratified by resistance classification.

Characteristics	MDR	XDR	PDR	Levene’s Test of Homogeneity (*p*)	ANOVA Test (*p*)
Age	70.29 ± 18.4	69.0 ± 14.7	61.1 ± 19.3	0.393	0.216
CPIS	6.3 ± 1.7	5.9 ± 1.7	6.1 ± 1.4	0.845	0.250
Charlson comorbidity index	5.4 ± 2.4	5.3± 2.1	4.3 ± 2.1	0.576	0.771
**Characteristics**	**MDR**	**XDR**	**PDR**	**Chi-Square Test (*p*)**	
Male, *n* (%)	3 (7.5)	26 (65)	11 (27.5)	0.585	
Having underlying disease, *n* (%)	7 (10.6)	42 (63.6)	6 (23.1)	0.113	

**Table 3:** Pathogenic, clinical and paraclinical characteristics of the patients in the study (*n* = 66).

*Pathogenic bacteria, n (%)*	
*A.baumannii*	45 (68.2)
*K. pneumoniae*	15 (22.7)
*P. aeruginosa*	6 (9.1)
***Clinical features***	
Fever, *n* (%)	43 (65.2)
Increased amount of endotracheal aspirated secretions, *n* (%)	45 (68.1)
Turbid endotracheal aspirated secretions, *n* (%)	66 (100)
Rales, *n* (%)	61 (92.4)
Fever, *n* (%)	43 (65.2)
***Paraclinical features***	
Leukocytosis, *n* (%)	49 (74.2)
PaO_2_/FiO_2_ ≤ 240, *n* (%)	37 (56.1)
Characteristics of infiltrates on chest X-ray, *n* (%)	
- 1 lung field	4 (6.1)
- 2 lung fields	25 (37.9)
- Scattered, around the hilia, indistinct	37 (56)

**Table 4:** Antibiotic resistance rate of multi-resistant GNB (*n* = 66).

Antibiotic Resistance Rate (%)						
Class	Subclass	Antibiotic	*Ab* ^1^	*Kb* ^2^	*Pa* ^3^	GNB
β-lactams	Penicillin	Amoxicilline/clavulanate	-	83.3	-	83.3
		Ampicillin	100	100	-	100
		Ampicilline/sulbactam	92.9	100	-	96
		Ticarcillin	100	100	100	100
		Ticarcilline/clavulanate	100	100	100	100
		Piperacillin	100	100	100	100
		Piperacillin/tazobactam	97.7	93.3	100	96.7
	Monobactam	Aztreonam	100	100	100	100
	Cephalosporin	Cefazoline	100	100	100	100
		Cefoxitin	100	100	-	100
		Ceftazidime	97.8	100	100	98.3
		Ceftazidime/avibactam	-	100	100	100
		Ceftriaxone	100	100	-	100
		Cefepime	97.7	100	100	98.5
	Carbapenem	Imipenem	97.8	86.7	100	95.5
		Ertapenem	-	80	-	80
		Meropenem	97.4	100	100	98
Aminoglycoside		Amikacin	78.6	50	80	70.4
		Gentamycin	86.7	86.7	83.3	86.4
		Tobramycin	84.2	71.4	80	82
Quinolone	Fluoroquinolone	Ciprofloxacin	100	100	100	100
		Levofloxacin	100	100	100	100
		Nitrofurantoin	-	100	-	100
Peptide	Polymixin	Colistin	2.8	-	20	4.9
Tetracycline	Glycylcycline	Tigecycline	-	0	-	0
		Minocycline	-	-	-	0
Phenicol		Chloramphenicol	-	50	-	50
Cotrimoxazole		TMP/SMX ^4^	64.3	73.3	-	66.7

^1^ *A. baumannii*, ^2^ *K. pneumoniae*, ^3^ *P. aeruginosa*, ^4^ Trimethoprim/sulfamethoxazole.

**Table 5:** Antibiotic regimens and combinations of colistin (*n* = 66).

Number of Antibiotics in Initial Regimens, *n* (%)	
one antibiotic	9 (13.6)
two antibiotics	53 (80.3)
three antibiotics	4 (6.1)
**Antibiotic Composition in the Initial Regimen, *n* (%)**	
Penicillin	12 (18.2)
Cephalosporin	15 (22.7)
Carbapenem	38 (57.6)
Amikacin	17 (25.8)
Fluoroquinolone	35 (53)
Glycopeptide	4 (6.1)
Oxazolidinone	4 (6.1)
Lincosamide	2 (3)
**Initial Regimen by Active Substance, *n* (%)**	
Initial regimen with carbapenem	38 (57.6)
Initial regimen with piperacilline/tazobactam	11 (16.7)
**Consistency of Initial Regimen, *n* (%)**	
with the recommendation of ATS/IDSA 2016	60 (90.9)
with antibiogram	4 (6.1)
**Change in Antibiotic Regimens when Antibiograms Were Available**	
Change in regimens (use of alternative ones)	52 (78.8)
***39/52 Combinations Containing Carbapenems***	
Combined with colistin	24 (61.5)
Combined with aminoglycosides	8 (20.5)
Combined with other antibiotics	7 (18)
***13/52 Combinations without Carbapenems***	
Combined with colistin	11 (84.6)
Combined with other antibiotics	2 (15.4)
**Features of Colistin Combination (*n* = 35)**	
Time of colistin use, mean ± SD, days	10.7 ± 5.7
**Route of Administering Colistin, *n* (%)**	
Intravenous alone	33 (94.3)
Intravenous + aerosolized	2 (5.7)
Aerosolized alone	0 (0)

**Table 6:** Treatment outcomes, acute kidney injury.

Content	Value (*n* = 66)
Response to the initial regimen, *n* (%)	43 (65.2)
7-day mortality, *n* (%)	4 (6.1)
28-day mortality, *n* (%)	36 (54.5)
Acute kidney injury, *n* (%)	8 (12.1)

**Table 7:** Twenty-eight-day treatment outcomes and related factors (*n* = 66).

Factors		28-Day Treatment Outcomes		OR (95% CI)	*p*
		Alive	Dead		
Characteristics of the Patients					
Underlying disease	Yes	26 (44.1%)	33 (55.9%)	0.591 (0.121–2.877)	0.693
	No	4 (57.1%)	3 (42.9%)		
Fever	Yes	16 (37.2%)	27 (62.8%)	0.381 (0.135–1.079)	0.066
	No	14 (60.9%)	9 (39.1%)		
Rales	Yes	27 44.3%)	34 (55.7%)	0.529 (0.082–3.388)	0.652
	No	3 (60%)	2 (40%)		
Leukocytosis	Yes	26 (53.1%	23 (46.9%)	3674 (1.05–12.865)	**0.049**
	No	4 (23.5%)	13 (76.5%)		
PaO_2_/FiO_2_ ≤ 240	Yes	17 (45.9%)	20 (54.1%)	1.046 (0.394–2.778)	0.928
	No	13 (44.8%)	16 (55.2%)		
Bilateral infiltrates/X-ray	Yes	9 (36%)	16 (64%)	0.536 (0.193–1.487)	0.228
	No	21 (51.2%)	20 (48.8%)		
*A. baumannii*	Yes	18 (40%)	27 (60%)	0.5 (0.175–1.429)	0.193
	No	12 (57.1%)	9 (42.9%)		
*K. pneumoniae*	Yes	9 (60%)	6 (40%)	2.143 (0.662–6.931)	0.198
	No	21 (41.2%)	30 (58.8%)		
*P. aeruginosa*	Yes	3 (50%)	3 (50%)	1.222 (0.228–6.553)	0.572
	No	27 (45%)	33 (55%)		
**Consistency of the Initial Regimens (*n* = 66)**					
with ATS/IDSA 2016	Yes	28 (46.7%)	32 (53.3%)	1.75 (0.298–10.29)	0.428
	No	2 (33.3%)	4 (66.74%)		
with antibiogram	Yes	2 (50%)	2 (50%)	1. (0.161–9.179)	0.621
	No	28 (45.2%)	34 (54.8%)		
**Change of Regimen (*n* = 66)**					
Yes		19 (36.5%)	33 (63.5%)	0.157 (0.039–0.634)	**0.005**
No		11 (78.6%)	3 (21.4%)		
**Carbapenem-Containing Regimen (*n* = 39)**					
Carbapenem + colistin		10 (41.7%)	14 (58.3%)	1.964 (0.483–7.989)	0.342
Carbapenem + others		4 (26.7%)	11 (73.3%)		
**Regimen with Colistin (*n* = 35)**					
Colistin + carbapenem		10 (41.7%)	14 (58.3%)	1.25 (0.287–5.449)	0.533
Colistin + others		4 (36.4%)	7 (63.6%)		
**Route of Administering Colistin (*n* = 35)**					
Intravenous only		13 (39.4%)	20 (60.6%)	0.650 (0.037–11.332)	0.647
Intravenous + aerosol		1 (50%)	1 (50%)		

Note: bold values show significant difference (*p* < 0.05).

**Table 8:** Multiple logistic regression with backward method (Wald) of 28-day outcome and independent factors.

Independent Factors (*n* = 66)		28-Day Outcome		
		HR	95% CI	*p*
Fever	Yes	0.309	(0.100–0.954)	0.041
	No (Ref)			
Leukocytosis	Yes	4.519	(1.194–17.101)	0.026
	No (Ref)			
	No (Ref)			

Note: variables entered in step 1: Fever, Leukocytosis, *A. baumannii*, *K. pneumonia*, Change of regimen, colistin combining.

**Table 9:** Cox model with hazards ratio of mortality and independent factors.

Independent Factors (*n* = 66)	HR	95% CI	*p*
Fever	0.511	(0.225–1.160)	0.108
Leukocytosis	1.828	(0.872–3.831)	0.110
*A. baumannii*	0.536	(0.150–1.908)	0.336
*K. pneumoniae*	0.944	(0.233–4.238)	0.994
Change of regimen	0.982	(0.427–2.257)	0.966
Colistin combining	0.788	(0.389–1.594)	0.507

**Table 10:** Acute kidney injury and related factors.

Factors	Acute Kidney Injury		OR (95% CI)	*p*
	Yes	No		
Type of Regimen (*n* = 66)				
Regimen with colistin	5 (14.3%)	30 (85.7%)	1.556 (0.34–7.121)	0.426
Regimen without colistin	3 (9.7%)	28 (90.3%)		
**Route of Administering Colistin (*n* = 35)**				
Intravenous only	4 (12.1%)	29 (87.9%)	0.138 (0.007–2.668)	0.269
Intravenous + aerosol	1 (50%)	1 (50%)		

## Data Availability

The datasets generated and/or analyzed during the current study are available from the corresponding author on reasonable request.
